# The Straw Pressure Gradient and Gravity (SPGG) Technique: A Safe and Cost-Effective Technique for Laparoscopic Suction

**DOI:** 10.7759/cureus.37779

**Published:** 2023-04-18

**Authors:** Farid Gerges, Elafra Nour, Ioannis N Gerogiannis

**Affiliations:** 1 Department of General and Emergency Surgery, Kingston Hospital NHS Foundation Trust, London, GBR

**Keywords:** drainage, intra-abdominal collection, laparoscopic technique, laparoscopy, suction

## Abstract

Suction devices are frequently used during laparoscopic surgery. However, their cost and limitations can be significant, depending on the clinical case, theatre setting and national health system. Furthermore, the continuous need to reduce the costs of the consumables in minimally invasive surgical procedures and their environmental burden creates extra pressure on the healthcare systems globally. Therefore, we present a new technique for laparoscopic suctioning, the Straw Pressure Gradient and Gravity (SPGG) technique. It is a safe, cost-effective and environmentally friendly technique compared to traditional suction devices.

The technique involves using a sterile, single-use Suction Catheter 12-16 French after positioning the patient according to the targeted collection. The catheter is inserted via the laparoscopic port nearest the collection and directed using laparoscopic graspers. The outer end needs to be clamped to avoid fluid spillage, and the catheter tip is placed in the collection. Then after the clamp is released, the fluid will be successfully drained due to the pressure gradient into a pot placed at a lower level than the intra-abdominal collection. Minimal wash can be performed via the gas vent by using a syringe.

SPGG is a safe and easy-to-learn technique that requires similar skills as placing an intra-abdominal drain during laparoscopy. It is softer than rigid, traditional suction devices and atraumatic. It can be used for suction, irrigation, collection of fluid for sampling and as a drain in case of an intraoperative indication.

SPGG is a cost-effective device as it is cheaper than the average disposable suction device system and has multiple uses, which can significantly decrease the annual cost of laparoscopies. It can also reduce the number of consumables and lighten the environmental burden of laparoscopic procedures.

## Introduction

Suction devices are frequently used during laparoscopic surgery. Most of those devices provide suction together with irrigation. They are designed to draw pooled fluid to clear the dissection field and wash out areas of the abdominal cavity to prevent postoperative intra-abdominal abscesses or collections [1]. However, as most of the suction/irrigation instruments used in laparoscopy are rigid and powered by a negative pressure device, there is a possibility of damaging soft and floppy intra-abdominal structures like the omentum and the small bowel [2-4]. In current and future global surgery, healthcare costs play a detrimental role. Therefore, cost awareness from all surgical specialities is crucial [5,6]. Especially with the increasing demand for minimally invasive surgery, there is a continuous cost analysis implemented by the healthcare systems globally. The cost reduction and standardisation of procedures to minimise the financial burden and, at the same time, maintain patients’ quality of care can be challenging [7].

Furthermore, the environmental impact of the suboptimal use of disposable consumables in surgery is significant, and there is a need for awareness by all healthcare professionals [8]. Therefore, we present a new technique for laparoscopic suctioning, the Straw Pressure Gradient and Gravity (SPGG) technique. It is a safe, cost-effective and environment-friendly technique compared to existing suction devices. It can also be used for minimal irrigation of the abdomen and for collecting intra-abdominal fluid for sampling.

## Technical report

SPGG is a simple technique based on some very well-known principles of Physics; Poiseuille’s Law, Pascal’s Law and the hydrostatic principle of communicating vessels [9].

During a laparoscopic procedure, the patient’s position is crucial for many reasons; One is to have better access and view of the targeted collection. Adjusting the patient, using the Trendelenburg versus the reverse Trendelenburg position with a combination of left or right axial rotation, will allow the intraperitoneal fluid to move along the abdominal quadrants. The gravity will trigger the fluid to move and stay in the lower part of the intra-abdominal cavity. The surgeon will then be able to target it with the SPGG.

**Figure 1:**
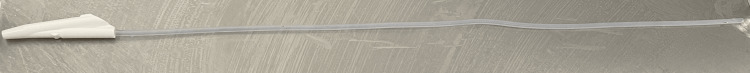
Single-use Suction Catheter. The plastic proximal end (white 12 French in this photo) should be cut and leave only the PVC part.

We use a sterile, single-use Suction Catheter 12-16 French (Figure 1).

**Figure 2:**
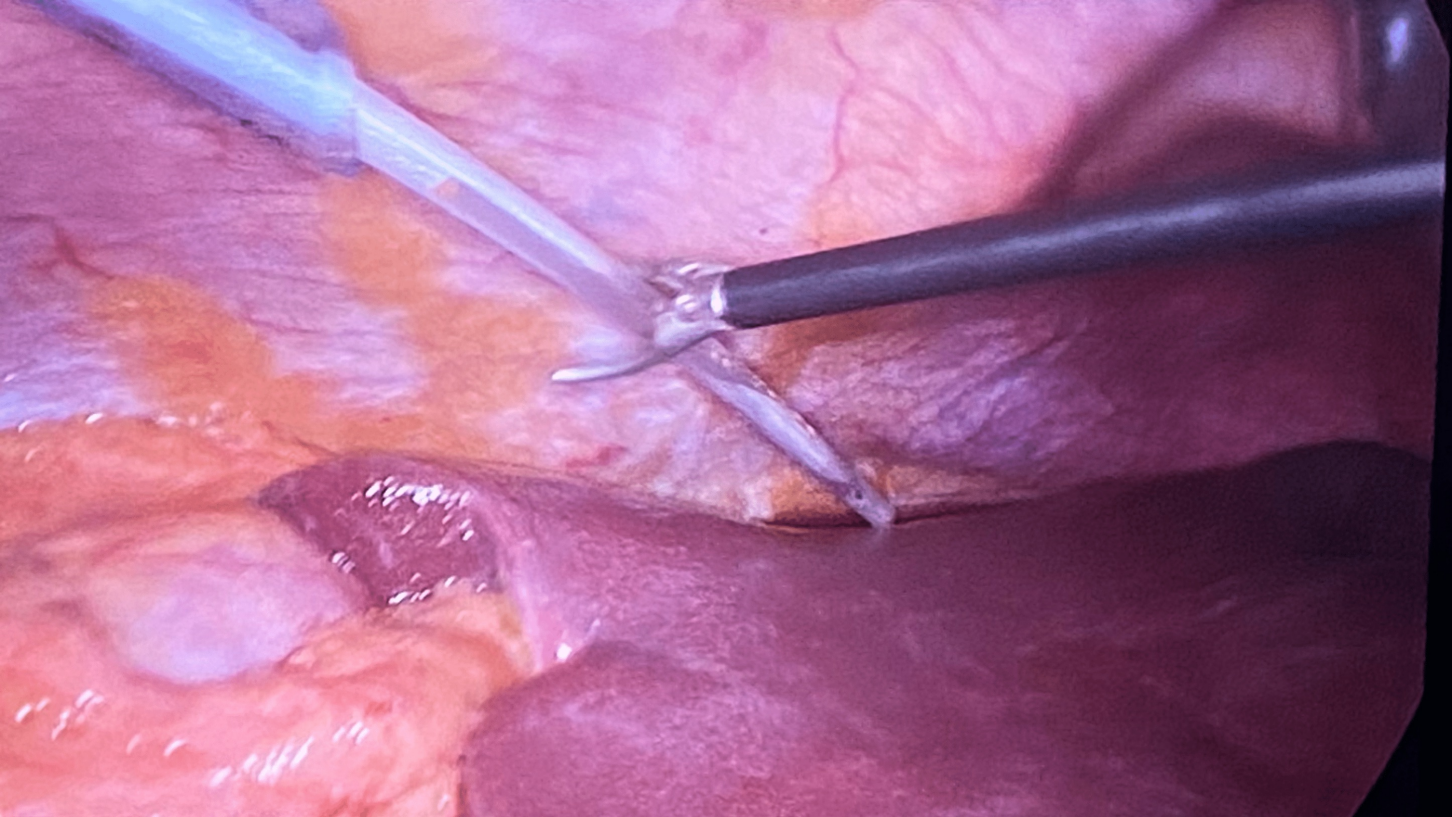
Laparoscopic view of the tip of the catheter which is guided to the collection above the liver, during laparoscopic cholecystectomy

The catheter is inserted via the port nearest the collection and directed using laparoscopic graspers (Figure 2).

**Figure 3:**
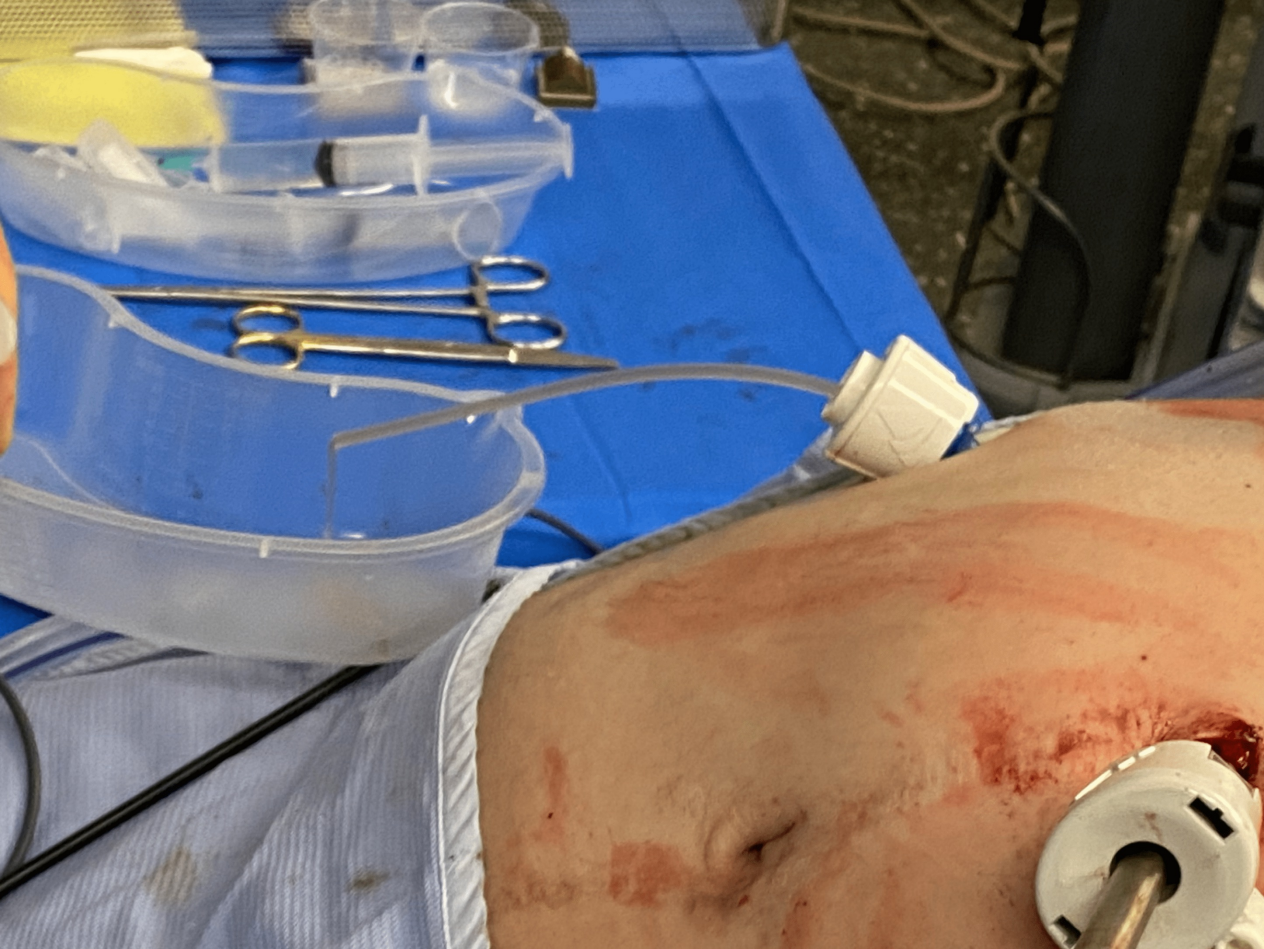
Pot used for the collection of the intraperitoneal fluid

The outer end needs to be clamped to avoid fluid spillage or loss of CO_2_. Next, the catheter tip is placed in the collection. Then, the clamp is released, and the fluid will be successfully drained due to the pressure gradient into a pot set at a lower level than the intra-abdominal collection (Figure 3).

**Figure 4:**
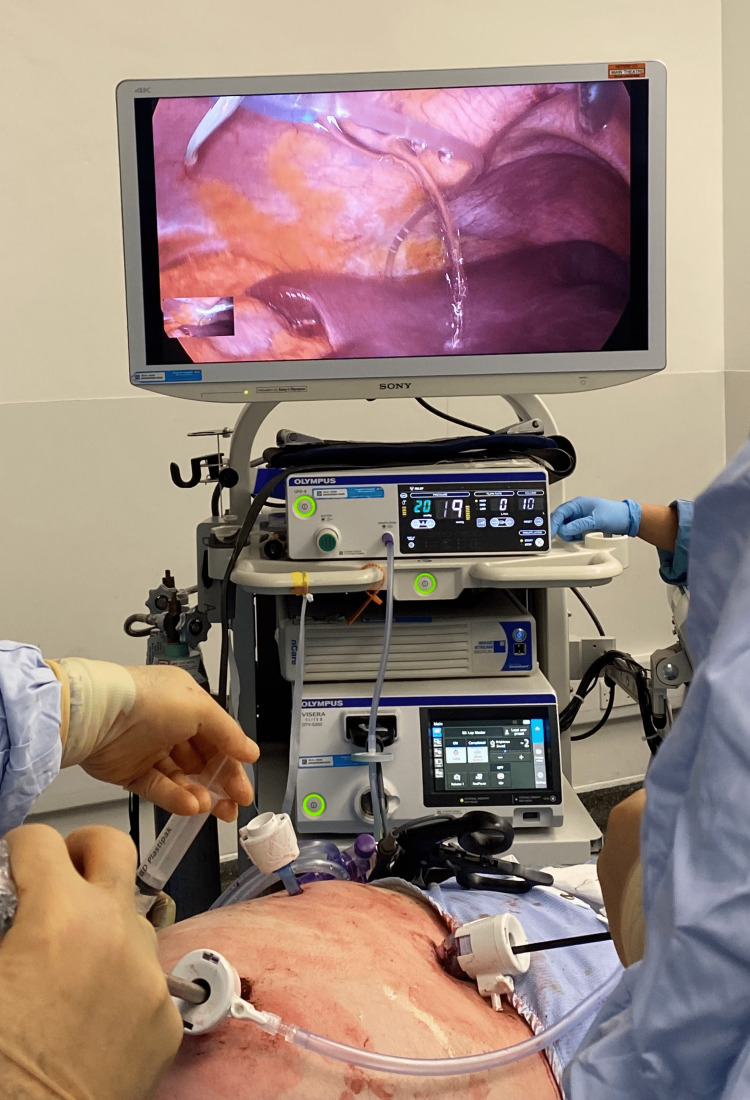
Intra-abdominal wash with a syringe via the port’s gas vent

Minimal wash can be performed via the laparoscopic port’s gas vent using a syringe with normal saline (Figure 4).

Finally, the catheter can be fixed with a silk stitch on the patient’s skin, in case there is a need for a drain of a size of 12-16 French, after removing the laparoscopic port at the end of the operation.

## Discussion

SPGG is a safe and easy-to-learn technique requiring similar skills as inserting an intra-abdominal drain during laparoscopy. Hence, all laparoscopic surgeons and trainees have the skills to use it.

The suction catheter is composed of medical-grade PVC and latex free. It is softer than the rigid traditional suction device and is atraumatic. The anaesthetic team mainly uses this catheter to clear the endotracheal tube or the oral cavity from secretions [10]. Therefore, this catheter can be found in any operation theatre or the Intensive Care Unit.

The intra-abdominal pressure (pneumoperitoneum) should be 12-15 mmHg for adequate drainage. Sometimes, intra-abdominal pressure may need to be increased by 2-6 mmHg for a few minutes to facilitate adequate fluid drainage. In that case, communication with the anaesthetic team is necessary to be aware and expect haemodynamic changes that might occur [11].

It is easy to learn and apply, similar to the skill set required to place an intra-abdominal drain during laparoscopic surgery [12]. The suction catheter is also more flexible than the stiff, traditional suction device; therefore, it can better accommodate the various contours of intra-abdominal organs. There is also less possibility of damaging the omentum or other intra-abdominal organs.

The fluid collected via the SPGG stays in the kidney dish and can be sampled separately and sent for analysis. The fluid drained using traditional suction devices ends up in the same canister as the irrigation fluid. This renders the intra-abdominal fluid unsuitable to be used as a sample for further laboratory testing unless another device is used and placed between the suction device and the canister. By using the SPGG, there is no need for extra devices for collection and sampling.

Limitations of the technique are that SPGG cannot be used to perform blunt dissection like the traditional rigid suction catheter or to perform rapid suction during critical situations such as managing or attempting to control active bleeding.

Surgeons play a pivotal role in reducing operating room expenses [6]. Using the SPGG is cost-effective for three reasons: (i) the SPGG is cheaper than the average disposable suction device systems (£0.7 versus £15), (ii) there is no need for an extra device to collect fluid for sampling and (iii) the catheter can also be used as a drain for post-operative care if needed.

Finally, from the environmental point of view, the SPGG technique uses significantly fewer disposable consumables even compared with the non-disposable suction devices if we take into consideration the use of disposable parts to connect with the negative pressure device.

## Conclusions

SPGG is a technique which is safe, efficient, cost-effective and easily applicable to all surgical specialities that use laparoscopy in any hospital and health system setting worldwide. Its main advantage is that it can decrease the annual cost of laparoscopies, making SPGG a technique that can be implemented anytime in any setting. In addition, the consumables of the SPGG device exist within the operation theatre, and there is no need for a formal business case or local approvals to obtain and use it. Finally, the technique can reduce the number of consumables and lighten the environmental burden of laparoscopic procedures. There is a need for future studies that will measure the cost-effectiveness of the SPGG compared with the traditional suction devices as well as the outcomes postoperatively.
