# Correction: The Straw Pressure Gradient and Gravity (SPGG) Technique: A Safe and Cost-Effective Technique for Laparoscopic Suction

**DOI:** 10.7759/cureus.c288

**Published:** 2025-09-09

**Authors:** Farid Gerges, Elafra Nour, Ioannis N Gerogiannis

**Affiliations:** 1 Department of General and Emergency Surgery, Kingston Hospital NHS Foundation Trust, London, GBR

The Ethics Statement and Conflict of Interest Disclosures section of this article has been corrected to indicate that this study did involve human subjects.

